# Treatment Response to a Double Administration of Constraint-Induced Language Therapy in Chronic Aphasia

**DOI:** 10.1044/2018_JSLHR-L-16-0102

**Published:** 2018-07-13

**Authors:** Jennifer Mozeiko, Emily B. Myers, Carl A. Coelho

**Affiliations:** a Department of Speech, Language and Hearing Sciences, University of Connecticut, Storrs

## Abstract

**Purpose:**

This study investigated changes in oral–verbal expressive language associated with improvements following 2 treatment periods of constraint-induced language therapy in 4 participants with stroke-induced chronic aphasia. Generalization of treatment to untrained materials and to discourse production was also analyzed, as was the durability of the treatment effect.

**Method:**

Participants with aphasia were assessed using standardized measures and discourse tasks at 3 to 4 time points to document behavioral changes throughout each of two 30-hr treatment periods of constraint-induced language therapy. Daily probes of trained and untrained materials were also administered.

**Results:**

Despite participant heterogeneity, behavioral results for each person with aphasia indicated a positive response to treatment following each treatment period indicated by performance on standardized tests, trained materials, or both. Treatment effects generalized to some degree to untrained stimuli and to discourse measures and were generally maintained at follow-up testing.

**Conclusions:**

Data support the utility of a 2nd treatment period. Results are relevant to rehabilitation in chronic aphasia, confirming that significant language gains continue well past the point of spontaneous recovery and can occur in a relatively short time period. Importantly, changes are not confined to a single treatment period, suggesting that people with aphasia may benefit from multiple doses of high-intensity treatment.

Intensive aphasia treatments may produce better outcomes than those administered less intensively (Brady, Kelly, Godwin, Enderby, & Campbell, 2016); however, treatment type, aphasia severity, and dosage specifics are all variables that contribute to outcomes and whose roles have yet to be disambiguated. Further complicating the situation, outcome measures must be sensitive to the many possible dimensions along which recovery may be manifested. In the current study, we initiate an exploration of some of the variables that impact responsiveness to treatment. Namely, we explore whether aphasia severity impacts responsiveness to treatment and whether a second treatment administration confers additional benefits.

Though typical outpatient speech-language therapy is administered at a session duration of about an hour and a session frequency of one to three times per week, treatments that tend to be flagged as effective in generating behavioral change, though not necessarily generalizability, are those delivered at higher intensities than that (Bhogal, Teasell, & Speechley, 2003; Cherney, Patterson, Raymer, Frymark, & Schooling, 2010; Kelly, Brady, & Enderby, 2010; Robey, 1998; Teasell et al., 2009). Fewer studies document generalizability and maintenance of gains, but of those that do, it has been demonstrated that short-term (1–2 weeks), high-frequency therapy (4–5 days per week) and long session duration (3 hr), resulting in 20–30 hr over 2 weeks, can result in stable improvements (Barthel, Meinzer, Djundja, & Rockstroh, 2008; Maher et al., 2006).

Current studies that claim to administer intensive treatment vary widely in their definitions of the dosage parameters of treatment (e.g., session duration and session frequency) and, thus, complicate interpretation of the contribution of intensity to treatment efficacy. Assumptions about the meaning of intensity stem from various literature reviews of intensively provided treatments. For example, Bhogal et al. (2003) found a significant immediate treatment effect for therapy administered at 8.8 hr per week over 11 weeks, whereas Robey (1998) made more general conclusions that a minimum of 2 hr of treatment per week was most effective. These reviews do not purport to define intensity; however, they are often referenced to justify use of the term *intensive* in any given treatment study. Just as a medication dosage is more than the number of pills to ingest (i.e., unit amount on the basis of patient weight, number of days to take medication, number of times a day to take medication), an aphasia treatment dosage is more involved than the number of hours of treatment administration.

Unfortunately, the terminology used in treatment literature is less straightforward than when applied to drug administration. For the purposes of this current study and so that other studies may be compared appropriately, we use intensity parameters initially proposed by Warren, Fey, and Yoder (2007), for children with intellectual disabilities, and modified by Cherney (2012), for use in describing aphasia treatment. Cherney (2012) describes a series of dose parameters, which, in combination, determine the intensity of the treatment. These parameters include session duration, session frequency, intervention duration, number of sessions, and the *dose* itself, which is defined as the number of teaching episodes per session. By multiplying dose by dose frequency by total intervention duration, it is possible to calculate a cumulative intervention intensity for any treatment (Warren et al., 2007). Because the number of teaching episodes were not tracked in the current study, we were unable to calculate a cumulative intervention intensity; however, the use of this metric by future researchers will make it easier to compare intensities among studies even when the individual dose parameters vary.

Months-long intervention durations, consisting of daily sessions and several hours-long session durations (Basso, 2001; Code, Torney, Gildea-Howardine, & Willmes, 2010; Mackenzie, 1991; Poeck, Huber, & Willmes, 1989) provide support that, unambiguously, high treatment intensity has positive immediate posttreatment outcomes reflected in standardized language test scores and qualitative analysis. Recently, high-intensity treatments of much shorter intervention durations and long session durations, such as constraint-induced language therapy (CILT; Barthel et al., 2008; Faroqi-Shah & Virion, 2009; Goral & Kempler, 2009; Maher et al., 2006; Mozeiko, Myers, & Coelho, 2011; Szaflarski et al., 2008), have also shown positive effects and authors consistently report maintenance of gains. Massed practice is one well-specified type of intensive treatment. CILT and others that utilize a massed practice approach provide high session frequency and long session duration over a relatively short intervention duration (1–3 weeks). The results are often lasting language gains that, in some instances, continue to increase after the completion of treatment (Barthel et al., 2008; Breier, Maher, Novak, & Papanicolaou, 2006; Maher et al., 2006; Meinzer, Djundja, Barthel, Elbert, & Rockstroh, 2005; Mozeiko, Coelho, & Myers, 2016). Other variables in the therapeutic process, specifically treatment type, also impact treatment outcome, making it difficult to separate out the contribution of intensity (Cherney, 2012). Melodic intonation therapy, for example, appears to have better results when administered intensively (Schlaug, Marchina, & Norton, 2009), whereas context-based treatments show no advantage with more intensity (Hinckley & Carr, 2005). We turn now to a discussion of one type of therapy that has often been applied in an intensive dose, CILT.

## CILT

CILT (also referred to as *constraint-induced aphasia therapy* or *intensive language action therapy*) requires forced use of the oral–verbal modality and was designed to be administered intensively (Difrancesco, Pulvermüller, & Mohr, 2012). Although versions of CILT have shown promise in terms of gains during treatment and persistence of those gains (e.g., Maher et al., 2006; Meinzer et al., 2005; Pulvermüller et al., 2001), it is not yet clear whether positive language gains are the result of the treatment type or the treatment intensity or some interaction between these factors. In one study that contrasted intensively administered CILT (30 hr over 2 weeks) with a distributed version of the same (30 hr over 10 weeks), benefits were apparent for participants from both groups, but changes in standardized scores were larger, and there was evidence of strong maintenance for those who received the intensive treatment with scores at or above immediate posttreatment scores on one or more measures (Mozeiko et al., 2016).

CILT is not unlike other evidence-based aphasia treatment programs that make use of principles guiding adaptive neuroplasticity. CILT targets the oral–verbal modality of language using shaping, scaffolding, and reinforcement. What makes CILT attractive for study is that, in each of the several studies in which it has been replicated, similar and well-defined dosage parameters have been reported (e.g., Breier et al., 2006; Johnson et al., 2013; Kurland, Pulvermüller, Silva, Burke, & Andrianopoulos, 2012). In addition, (a) positive language gains for a range of aphasia types have been reported, (b) the short duration provides logistic feasibility for researchers and participants, and (c) it provides experimental control across studies insofar as the participants within each dyad experience identical treatment conditions. For these reasons, CILT lends itself as an appropriate treatment for which to begin an investigation of intensity parameters and potential change in effect when these parameters are manipulated.

## Treatment Population: Mild Aphasia

An important variable to consider when selecting an aphasia treatment is the severity of the disorder. Still unresolved is whether intensive treatments are appropriate for people with mild aphasia types (Western Aphasia Battery Aphasia Quotient [WAB-AQ] 75–93.8; Kertesz, 1982). Sage et al. (2011) reported that two groups of participants with anomia made equal gains following intensive versus nonintensive sessions of the same cueing therapy and that gains were maintained better for those who received the nonintensive therapy. Anomia is a diagnosis often considered a milder aphasia type, as classification according to the WAB requires relatively higher scores on fluency, verbal comprehension, and repetition, but aphasia severity may be more moderate (WAB-AQ < 75) if greater deficits in word finding and naming are demonstrated. Anomia diagnoses were not quantified in the aforementioned article, but results call into question whether intensive therapies bring about the same gains in milder aphasia types as have been reported in more moderate to severe cases. Indeed, CILT has been used for those with a range of aphasia deficits, including those with mild aphasia, but gains for this population are reported to be the most limited. Most studies using CILT include only people with moderate–severe aphasia (Breier et al., 2006; Johnson et al., 2013; Maher et al., 2006; Szaflarski et al., 2008; Wilssens et al., 2015). In one study, CILT was modified to focus on grammaticality for participants with aphasia ranging in severity from moderate to mild (pretreatment WAB-AQ range = 62.9–93.7; Faroqi-Shah & Virion, 2009). Despite reports of positive performance on tests of morphosyntax, limited changes were reported for aphasia severity, and the two participants with lower initial scores were those who demonstrated larger changes after treatment. In larger group studies that included participants with mild aphasia, individual data are often not presented (e.g., Berthier et al., 2009; Pulvermüller et al., 2001).

Meinzer et al. (2007) analyzed data from several of their own studies using CILT and reported that 38 of 44 people made improvements on standardized tests and that, although results were not correlated with aphasia chronicity nor with age, they were correlated with initial severity of aphasia as indicated by the Aachen Aphasia Test (Huber, 1984) profile score. The authors attributed their findings to the learned non-use hypothesis, positing that those who have withdrawn from verbal communication the most might derive the greatest benefit from CILT.

Given that the majority of participants in studies of CILT are moderately to severely impaired, it is possible that the materials used in those studies were not sufficiently challenging to those with milder aphasia types. CILT is described as requiring, minimally, an attempt on a high-frequency noun *(book)* and, at the most, a full sentence, including a carrier phrase (“Do you have the book?”). Maher et al. (2006) created a more challenging version of CILT by introducing a hierarchy that included decks with semantically similar cards, decks requiring the use of an adjective *(blue book)*, and decks requiring two adjectives *(two blue books)* for participants with moderate aphasia. In sum, producing and responding to simple sentences may not provide the challenge necessary to instantiate change in people with mild aphasia.

When change does occur, gains may not be evident for people with mild aphasia if standardized tests are the only measure of change as they are inadequate for participants performing near ceiling. Discourse production and comprehension tasks are examples of more sensitive methods of documenting functional change, particularly for those with mild aphasia symptoms. Few studies using constraint-induced-type treatments have used discourse production as an indicator of change (e.g., Maher et al., 2006; Rose, Attard, Mok, Lanyon, & Foster, 2013).

## Treatment Intensity: Two Treatment Periods

Treatment intensity is often couched in terms of number of hours per sessions *(session duration)* or sessions per week *(session frequency)*. Total treatment duration is also relevant and, in CILT, varies between 1 to 4 weeks (e.g., Bakheit et al., 2007; Szaflarski et al., 2015); in studies that use a 15-hr per week schedule, 3 weeks is rarely exceeded. Of interest is whether continuing treatment beyond the usual 2-week treatment period confers additional benefits to persons with aphasia (PWAs). Different methods and outcome measures make it difficult to compare definitively among studies but do provide suggestive evidence that more is better than less. For example, a 3-week program by Johnson et al. (2013) resulted in 10-plus point changes on the WAB-AQ for three out of four participants, and these results appear more robust than those of a 1-week program (Szaflarski et al., 2008) in which Boston Diagnostic Aphasia Examination (BDAE; Goodglass & Kaplan, 2000) scores increased by less than 2 points for most participants. It is not clear, however, that 3 weeks confer more benefit than other 2-week regimens. Three of four participants in each of two 2-week programs, one by Maher et al. (2006) and one by Mozeiko et al. (2016), made gains greater than 6 and 8 points, respectively, on the WAB-AQ. It remains uncertain as to whether an additional treatment period would produce continued improvements.

The most intensive treatments exceeding 3 weeks tend to include a combination of therapies—for example, a combination of group, computer, and individual treatment (e.g., Babbitt, Worrall, & Cherney, 2015; Code et al., 2010). Analysis of two Intensive Comprehensive Programs (ICAPS) provided at 23–25 hr per week for 4–6 weeks revealed that 45 out of 70 participants made at least a 5-point gain on the WAB-AQ, suggesting positive effects (Persad, Wozniak, & Kostopoulos, 2013). It is not clear whether these effects are greater than what would be seen following just 2 weeks of the same treatment. Importantly, it remains to be seen whether gains following more than 100 hr of ICAPS exceed those followed by 30 hr of CILT because studies demonstrate equally positive results.

Finally, the effects of two different high-dosage treatments on a single individual have been compared in recent studies (Kempler & Goral, 2011; Kurland et al., 2012; Rose et al., 2013). In each, two different treatments were administered in equal time blocks one after the other in order to compare effects. In each, a continued positive effect was observed, though some effects appeared to be attenuated compared to those seen following the first treatment. For example, a participant in a study comparing CILT to multimodal aphasia therapy (Rose et al., 2013) increased in accuracy on trained nouns by approximately 280% following multimodal aphasia therapy. Little additional change in performance was observed following the second treatment with CILT. One participant who received CILT first, however, increased in percent accuracy by approximately 355% on this same measure but not higher than this after multimodal aphasia therapy, administered second. This points to a potential order effect, suggesting that the largest gains are likely to be observed following initial treatment phases. A period of no treatment separating the two treatments may have allowed for the consolidation or decay of gains following the first treatment period and may have then better illustrated the effect of each period on its own. It is also possible that continuing with a single treatment may have led to increased effects as it would have provided more time for mastery and to build on newly developed skills.

Although the main benefit of a double administration of a single treatment is predicted to be an increase in gains on trained materials, there are other potential advantages. One is to determine whether a maximum treatment effect is reached during this period—a point at which gains plateau despite continued intensity. Second is the potential for better maintenance of gains. The effect of hundreds of task repetitions to achieve automaticity has been documented in the motor literature (e.g., Nudo & Milliken, 1996). This concept of extended practice well past the point of mastery is not as well documented in the aphasia literature, but implications are positive (Kurland et al., 2007).

## Generalization of Target Behavior

*Generalization* refers to a transfer of skills to environments outside the clinic setting and improvements in behaviors not targeted during treatment. Despite the fact that generalization has been deemed the “gold standard in treatment research” (Thompson & Shapiro, 2007, p. 37), it is inconsistently reported in aphasia treatment studies.

CILT is considered a treatment of verbal expression, and when generalization is measured, it tends to be in terms of untrained verbal productions, such as in narrative discourse (Maher et al., 2006) or untrained words (Kurland et al., 2012). Increases in these areas suggest that this treatment has an effect on a host of language skills and has important implications for the future of treatment and treatment research. Studies that report individual data also show evidence of transfer to untrained verbal comprehension after treatment. For example, in studies that reported standardized battery subtests prior to and post CILT (e.g., Breier et al., 2006; Mozeiko et al., 2016; Szaflarski et al., 2008), notable gains in spoken language comprehension were recorded. Generalization resulting in changed scores in reading, writing, and cognition as a result of CILT has yet to be reported. Careful documentation of changes in all communicative domains and cognitive underpinnings could benefit future treatment of verbal comprehension in chronic aphasia.

## Summary of Problem

There is evidence that increasing the duration of high-dosage treatments may yield positive effects in language behavior that are both durable and generalizable to functional verbal language (Bhogal et al., 2003; Cherney, Patterson, Raymer, Frymark, & Schooling, 2008; Robey, 1998). Positive reports appear to be particularly consistent after utilization of the massed practice schedule approximating 30 hr over 2 weeks (Barthel et al., 2008; Meinzer et al., 2005). Nonetheless, the most recent review of treatment studies that compares differing dosages reported no clear differences between intensive and nonintensive treatments across studies (Cherney, Patterson, & Raymer, 2011). Equivocal results are likely attributable to lack of definition in what constitutes intensity. In order to determine optimal dosage parameters, each parameter must be manipulated within various treatment protocols for various patient populations. In the current study, we sought to increase the potential gains that result from massed practice for two people with mild and two with moderate–severe aphasia by administering CILT twice. During this time, language changes were programmatically assessed using multiple outcome measures. Specific experimental questions were as follows:

1. What is the effect of CILT for trained and untrained material after one and two treatment periods (each treatment period = 30 hr over 2 weeks)?
2. Will treatment effects be maintained at follow-up assessment 8 weeks after treatment completion?
3. Will aphasia severity influence performance in terms of treatment accuracy, maintenance, and generalization to discourse and untrained materials?

## Method

### Participants

Six participants were recruited from a university aphasia group on the basis of interest in the study and willingness to commit time for all assessments, treatment periods, and follow-up testing. All participants provided informed consent prior to initiation of the study, approved by the university's institutional review board. Inclusion and exclusion criteria included single, left-hemisphere stroke at least 12 months prior to the study; monolingual, native English speaker; right-handed; no reported history of psychiatric illness or acute, unstable medical conditions; ability to name at least two items on the Boston Naming Test (BNT; Kaplan, Goodglass, & Weintraub, 1983); normal or corrected hearing and vision; demonstrated understanding of the study; and ability to provide informed consent. Two participants were included who did not meet all inclusion criteria—one had aphasia as a result of an anoxic event, and one was left-handed. They were included because it was believed that they would benefit from the treatment and because two additional participants were needed in order to create the desired two triads. For the purposes of this study, only the findings from the four participants who met inclusion criteria will be discussed.

Though the protocol was originally designed and tested with both triads and dyads (Pulvermülle et al., 2001), there are important reasons to consider one over the other. Using dyads, each participant can receive overall more teaching episodes (in this case, more opportunities to request and respond to stimuli)—in other words, in a larger dose according to definitions by Warren et al. (2007) and Cherney (2012). When at least three people are involved, however, the game aspect is both more difficult and more interesting to those playing. The goal is to collect matching picture cards, and if there are only two participants, there is no game; there is only the requesting of pictures, and a win is the result of luck. By involving a third participant, the person requesting the card also has the additional cognitive task of remembering who has the card he or she is seeking. Given the length of the proposed study (a total of 60 hr of CILT), it was determined that maintaining participant interest was critical; therefore, increased repetitions were sacrificed.

Demographic data for each of the four participants appear in Table 1. Three male participants and one female participant ranged in age from 47 to 79 years. All participants attained at least a high school–level education. Three were employed prior to their stroke, and one had retired. Participants were between 31 and 58 months post onset. Two participants, M1 and M2, were classified by Western Aphasia Battery–Revised Aphasia Quotient (WAB-R AQ; Kertesz, 2006) scores as fluent anomic (95 and 87.6, respectively). Error patterns differed between these two participants (M1 and M2), but both demonstrated word-finding difficulty and occasional paraphasias but generally good grammaticality, good repetition, adequate auditory comprehension, and functional reading and writing skills. The participants with severe language deficits did not fit neatly into a specific aphasia classification. One participant, S1, had a severe nonfluent aphasia (WAB-R AQ = 38.5). Although he scored in the 90th percentile for accuracy on the apraxia subtest of the WAB-R, he did demonstrate speech characteristics consistent with apraxia of speech, including distorted sound substitution errors and multiple unsuccessful attempts to correct errors for spontaneous language (Duffy, 2005, p. 95), so apraxia of speech could not be ruled out. This was not believed to have interfered with S1's ability to produce adequate responses during treatment. The fourth participant, S2, had moderate–severe fluent aphasia (WAB-R AQ = 51.7) characterized by normal prosody and strings of grammatically appropriate jargon interspersed with actual content words and with both phonemic and neologistic paraphasias.

**Table 1.** Participant characteristics.

Characteristic	M1	M2	S1	S2
Age (years)	54	47	56	79
MPO	58	57	51	31
Sex	M	M	M	F
Handedness	R	R	R	R
Hemiplegia	moderate–severe	none	moderate	mild arm monoplegia
Occupation	Owner, steel fabrication company	Treasury project manager	Mechanical engineer	Insurance company purchasing office
Education (years)	16	16+	16	12
BNT	92%	77%	5%	5%
RCPM	89%	87%	95%	49%
WAB-R AQ	95.0	87.6	38.5	51.7
Spontaneous speech	95%	95%	35%	65%
Auditory verbal comprehension	100%	91%	85%	71%
Word fluency	65%	50%	10%	10%
Object naming	93%	97%	40%	60%
Reading	100%	96%	46%	44%
Writing	80%	88%	25%	52%
Language production	Fluent anomic. Slow, deliberate, often circumlocutory speech.	Fluent anomic. Effortful speech marked by hesitations, incorrect word choice, multiple self-corrections.	Severe nonfluent. Uses overlearned phrases and entrenched stereotype. Mild AOS.	Moderate–severe fluent. Long sentences, normal prosody, little intelligible content.

*Note.* Test scores are presented as percent correct with the exception of the WAB-R AQ for which the raw score is presented. MPO = months post onset; M = male; F = female; R = right; BNT = Boston Naming Test (Kaplan, Goodglass, & Weintraub, 1983); RCPM = Raven's Coloured Progressive Matrices (Raven, Raven, & Court, 2004); WAB-R AQ = Revised Western Aphasia Battery**–**Aphasia Quotient; AOS = apraxia of speech.

### Experimental Design

A modified multiple baseline design across participants was used in conjunction with a multiple probe technique to evaluate the effects of treatment (Thompson, 2006).

#### Treatment Level Establishment

Prior to treatment, probe testing was completed for each level in the treatment hierarchy to determine starting level of treatment. During probe testing, participants were shown 20 stimuli from each of eight predetermined treatment levels and asked to respond to each after having been provided with a model for each level. Details on levels of treatment and scoring of treatment probes at each level are discussed in Appendix A. It was determined that treatment would begin at the lowest level for which there was a less than 80% accuracy rate for two consecutive probe sessions. If participants within a triad tested at different levels, treatment would start at the level calibrated to the most impaired participant. For example, if one person scored 85% on Level 4 and another scored 75%, treatment would begin at Level 4 and would not move to Level 5 until all participants achieved at least 80% accuracy. This testing, which will be referred to as treatment level establishment (TE) going forward, occurred only for participants starting above Level 1, and this was completed prior to baseline testing.

#### Baseline Probes

Baseline probes were then conducted on the starting level of treatment until a pattern of stability emerged. *Stability* was defined as three consecutive data points not exceeding a 10% change from the first of the three. Using these criteria, between four and six baseline probes were necessary depending on the participant in order to establish a stable baseline. Baseline probes were conducted within a 2–5-week time span per participant and were always conducted a minimum of 48 hr apart. In order to maximize experimental control, a typical multiple baseline design across participants design introduces treatment sequentially, such that one participant will start treatment and baseline testing continues for others (Thompson, 2006). This demonstrates that behavior is only affected when treatment is applied but was not possible in the current study given the small group treatment design. The multiple probes allow for investigation of performance on increasingly difficult linguistic targets over the duration of the treatment period.

All participants also received baseline probes of productivity of discourse production on the same days they received baseline treatment probes. Baseline probes of discourse were meant to help characterize performance over time, but lack of stability did not preclude a start to treatment, as the primary outcome measure was the trained stimulus items.

#### Probes of Treated and Untreated Materials

Treatment probes identical to those used in baseline testing were administered prior to each training session starting after the second day of CILT. The treatment probes consisted of (a) 20 trained stimulus items—those that were included in previous treatment sessions, (b) 20 equivalent untrained stimulus items, (c) 20 items from the previously trained level to track maintenance, and also (d) 20 stimulus items from the subsequent level, the latter serving as continued baselining until training commenced at that level.

#### Discourse Production Probes

Each participant was shown three Norman Rockwell illustrations and asked, “Tell me what's happening in this picture,” for each. Each data point calculated reflects an average of the output for the three samples elicited in each probe. *Productivity* refers to the quantity of information and is based on the number of correct information units (CIUs) within a sample (Brookshire & Nicholas, 1994). CIUs are intelligible, nonrepeated words that are relevant to the illustration but not necessarily grammatically accurate. The number of CIUs was averaged across the three illustrations resulting in one data point. Efficiency and informativeness were calculated from the productivity measure (Nicholas & Brookshire, 1993) for each narrative and then averaged to create one baseline probe. *Efficiency* takes into account the speed of production (CIUs per minute). *Informativeness* provides a measure of relevant information conveyed as a proportion to all output (CIUs: total word count [TWC]). Discourse production was probed starting the day after the commencement of treatment probes and on alternate days throughout treatment. Productivity was an outcome measure of interest for the participants with moderate to severe aphasia. Efficiency and informativeness were of more relevance for mild participants capable of producing complete descriptions but who implemented long or multiple pauses or made use of circuitous language. (See Appendix A for protocol on treatment and discourse production probes).

#### Timeline

Once baselines were established and pretreatment testing was complete, Treatment Period I commenced with a triad of participants seen together for a 3-hr session, 5 days per week, for 2 weeks. This was followed by a set of probes for each treatment level (mirroring the TE procedure), discourse probes, and the administration of the WAB-R AQ and BNT. Participants received no treatment for the next 5 weeks. Following the no-treatment period, probes of trained and untrained materials and probes of discourse production were again administered for each treatment level, and then, Treatment Period II was conducted for another 2 weeks, followed by 2 days of posttreatment testing. Final assessments took place 8 weeks after the completion of Treatment Period II.

The 8-week follow-up period was chosen based on studies using CILT that consistently demonstrated maintenance of gains 4 weeks post treatment (Johnson et al., 2013; Maher et al., 2006; Mozeiko et al., 2016; Szaflarski et al., 2008). Maintenance at time points long after treatment completion is more suggestive of permanent change, but fewer studies report maintenance data beyond a month's time. Eight weeks is not long enough to determine whether changes will be longstanding but was determined to be more informative than a 4-week follow-up, with minimal increased risked of participant attrition.

#### Standardized Tests

The WAB-R AQ and the BNT were each administered four times: pre–Treatment Period I and post–Treatment Period I, post–Treatment Period II, and 8 weeks following treatment completion. The Computerized Revised Token Test (CRTT; McNeil et al., 2015) and the remainder of the WAB-R were administered three times to measure potential changes in cognitive function, comprehension, reading, and writing ability. Testing between treatment periods on these secondary measures was not performed.

Changes on related functions as a result of oral–verbal language stimulation treatment were tracked to increase understanding of the impact of CILT. Because these tests were used to measure change and repeated three or four times, internal consistency, standard error of measurement (SEM), determination of change signifying clinical significance, and test–retest reliability were important considerations. The WAB-R yields an aphasia quotient (AQ) used as a measure of severity and is a validated, standardized aphasia assessment reported in numerous studies (Bakheit et al., 2007; Kiran, Sandberg, & Sebastian, 2011; Thompson, den Ouden, Bonakdarpour, Garibaldi, & Parrish, 2010). Shewan and Kertesz (1980) report good test–retest reliability (*r* = .88, *p* < .001) and internal consistency (*r* = .974) on this measure.

The complete WAB-R was administered three times: pre–Treatment Period I, post–Treatment Period II, and 8 weeks following treatment. Only those subtests comprising the AQ score were administered post Treatment Period I as it was in these subtests that changes were anticipated. A 5-point increase on the AQ tends to be used as the benchmark indicating clinical significance (Shewan & Kertesz, 1980), though results of Rasch analysis suggested a variable SEM according to aphasia severity (> 2 points for AQs 30–70 ranging > 6 points for AQs < 20 and > 90; Hula, Donovan, Kendall, & Gonzalez-Rothi, 2010). The 5-point benchmark was used in this study.

The BNT (Kaplan et al., 1983) is a 60-item confrontation naming test and was included as an additional measure of untrained spontaneous naming. No SEM has been reported for the BNT for PWAs, but Flanagan and Jackson (1997) reported an SEM of 1.02 for individuals with no brain injury. The CRTT (McNeil et al., 2015) was used as a more sensitive measure of auditory comprehension than subtests of the WAB-R. McNeil et al. (2015) reported an overall SEM of 0.25–0.35 on this battery with test–retest reliability on subtests ranging from .79 to .91 and .85 overall. The CRTT was used due to reports from previous studies using CILT, indicating that much of the change on pretreatment to posttreatment WAB-R AQ scores can be attributed to changes in receptive language (Johnson et al., 2013; Mozeiko et al., 2016; Szaflarski et al., 2008). Unless otherwise specified, a 2-*SD* change on normed tests or a 20% change on nonnormed tests is considered clinically significant (Robey, Schultz, Crawford, & Sinner, 1999).

### Treatment Protocol

CILT was administered over two treatment periods for a total of 60 hr of treatment. It began at Level 1 for participants S1 and S2 and with Level 4 for M1 and M2 (see Appendix B for details on treatment stimuli used for each level). Each period consisted of 30 hr of treatment over 2 weeks with a 5-week break in between. Treatment began every morning following daily treatment probes. CILT uses an interactive game approach, following the rules of the card game Go Fish. Each participant was offered a cardholder in the event that hemiparesis precluded independent control and shielding of cards. Each was dealt five to seven cards, depending on familiarity with the deck, and was then instructed to request matching cards from other players. Participants were asked to respond as completely as possible. Requests and responses were initially modeled by the clinician. Once the participant demonstrated understanding, modeling was discontinued unless the client indicated a need for help. The clinician also cued responses with either phonemic or semantic information when necessary and reminded participants to use only the verbal modality of communication as needed. Following the constraint recommendations of CILT, written communication was disallowed, and gesture was only permitted as an accompaniment to verbal production but not as a substitution for a word or phrase. For example, holding up two fingers to symbolize the number 2 or pointing to a shirt instead of saying the word was unacceptable, but hand waving or gesticulating was permitted.

The constraint aspect of this treatment (i.e., constraint solely to the verbal modality of language) has been a point of debate among researchers because the term infers restraint of gesture, and there is evidence of a facilitatory nature of gesture in speech production. Difrancesco et al. (2012), however, provided a detailed explanation of constraint as it was originally conceptualized for this language therapy, dispelling the implication of restrained hand movements. Nonspecific gestures or hand movements accompanying verbal language were permitted, in keeping with these guidelines. Each participant was actively involved, and both produced and responded to requests for the full 3 hr. Participants were provided a 10-min break after the first 90 min.

If it was thought that the participant might not be able to produce the utterance as modeled by the clinician, a semantic or phonemic cue was provided prior to the production in order to reduce the production of errors (Maher et al., 2006). Cues were faded over time as independent, accurate productions became more consistent. Once mastery of the targeted materials was achieved by all participants in the group as demonstrated with > 80% accuracy on treatment probes, a new deck requiring a more complex response was introduced. A treatment hierarchy was designed with the protocol described by Maher et al. (2006) in mind; however, additional levels (described in Appendix B) were created in an attempt to challenge the participants with least impairment and also in anticipation that more levels would be mastered with the addition of the second treatment period.

Starting level was based on individual performance during the period of TE, prior to baseline testing. Points were awarded based on production, and a minimum of 80% of all possible points per level were necessary before progressing to the next level. For example, because Levels 1 and 2 required the accurate naming of 20 nouns, 1 point was scored per noun, so 16 of the 20 stimuli items had to be named correctly. Subsequent levels requiring additional words were assigned a greater number of points. The carrier phrase required in Level 3 was awarded 1 additional point per stimulus item. The addition of adjectives in Levels 4 and 5 meant an addition of 1 point per adjective. Prepositions were assigned 1 point each in Levels 6 and 7. All points were totaled in the end and divided by the maximum number of points possible to attain a percent correct score. In Level 8, the total number of CIUs produced per utterance was divided by the total number of words in order to calculate an equivalent percent correct score for that level. (Details of scoring at each level can be found in Appendix B).

If after 1 week (15 hr of treatment), Level 1 (high-frequency objects) mastery was not achieved, Level 2 (low-frequency objects) was introduced and trained simultaneously. This was done in order to ensure exposure to a greater number of stimuli without a negative consequence to word learning (see Snell, Sage, & Ralph, 2010). Importantly, this exposure was also felt to be consistent with the goal of maximizing and maintaining the interest and engagement of the participants. It was predicted that all participants would progress, such that moving forward prior to meeting criterion would not be necessary. This turned out not to be the case for the participants with severe aphasia, however.

**Figure 1.**
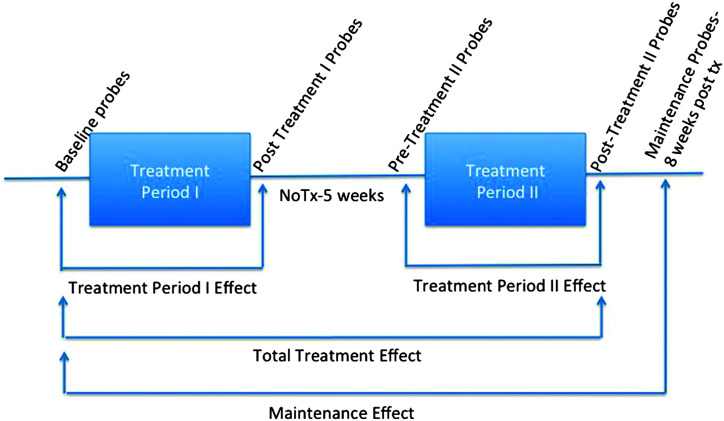
Depiction of effect size periods (Treatment Period I effect, Treatment Period II effect, total treatment effect, follow-up effect). Tx = treatment.

Following Treatment Period I, participants received probes at all treatment levels and 1 week of posttreatment testing followed by no treatment for 4 additional weeks. Five weeks post Treatment Period I, probes were administered again just prior to Treatment Period II. Performance on probes 1 week and 5 weeks post treatment was assessed for maintenance or change and to determine the starting level of Treatment Period II. If performance during the no-treatment period increased on the level being probed to at least 80% accuracy for all participants, the subsequent level would be treated once treatment recommenced (see Figure 1 for timeline.) Participants who did not reach 80% accuracy on Level 1 by this point would still move to Level 2 to ensure stimuli exposure and to maintain interest, as described above.

The risk of including a period of no treatment was stopping on the verge of a participant's potential mastery. The determination for the 5-week no-treatment period, however, was driven by current findings reported post CILT, which state that, for some participants, continued language gains occur up to a month post treatment (Johnson et al., 2013; Maher et al., 2006; Mozeiko et al., 2016; Szaflarski et al., 2008).

#### Treatment Data Analysis

Results of the study were examined on an individual basis, in keeping with single-subject experimental design conventions (Beeson & Robey, 2006). There is no general agreement on the best means of analysis for research of single-subject experimental design. Visual inspection, trend lines, binomial tests, analysis of variance, the C-statistic, standardized effect sizes, and clinical significance—often defined as a 2 *SD* change on standardized tests or by 20% on nonstandardized measures—have each been used to describe the effects of aphasia treatment. Each has its strengths and each its limitations (Robey et al., 1999), and even those deemed necessary, such as visual inspection, have questionable validity on their own. As such, responsiveness to treatment was examined based on a combination of measures. These included change in performance on standardized tests, dependent variables, slopes, effect sizes for trained and untrained materials, and effect sizes for discourse production.

The intensive nature of this treatment was expected to stimulate neural activation. By inducing the use of hundreds of words, including those that the PWA was likely to avoid, it was anticipated that inactive or dysfunctional system processes would become reengaged, and thus, treatment was expected to result in gains beyond the trained stimuli to untrained stimuli and also connected speech. Because increased, more efficient verbal language production is the ultimate goal of treatment and not simply mastery of a set word list, the generalization measure of connected speech also served as a main outcome measure. Discourse probes and all Level 8 probes were transcribed and analyzed by trained research assistants who were blinded as to when the samples were collected. Point-to-point intrarater and interrater reliability was performed by the first author for CIU analysis and ranged for intrarater reliability from 95.4 to 97.2. Interrater reliability was 94.3.

Effect sizes for performance on discourse production and trained stimuli were calculated using Busk and Serlin's (1992) variation on Cohen's *d* statistic as presented by Beeson and Robey (2006) in order to avoid the Type I error that may occur with visual inspection alone. For studies of naming, the benchmarks recommended by Robey and Beeson (2005) are 4.0, 7.0, and 10.1, corresponding to small, medium, and large effects, respectively. It should be noted, for comparison purposes, that recent aphasia treatment studies continue to use benchmarks of 2.6, 3.9, and 5.8 from Robey et al. (1999), which are based on single-subject aphasia treatment studies but not specifically those of lexical retrieval. The former, more conservative benchmarks were used in the current investigation. Effect sizes for follow-up were also calculated by comparing baseline to follow-up probes as detailed by Bailey, Eatchel, and Wambaugh (2015). These were then compared with immediate posttreatment effect sizes to help determine whether there was maintenance of gains.

Generalization to reading, writing, and cognitive functioning as measured by the Raven's Coloured Progressive Matrices (RCPM; Basso, Capitani, & Laiacona, 1987) and cortical quotient was not anticipated, but testing of each was included in the three assessments to gauge whether 60 hours of treatment might result in generalization to these related language processes.

## Results

**Figure 2.**
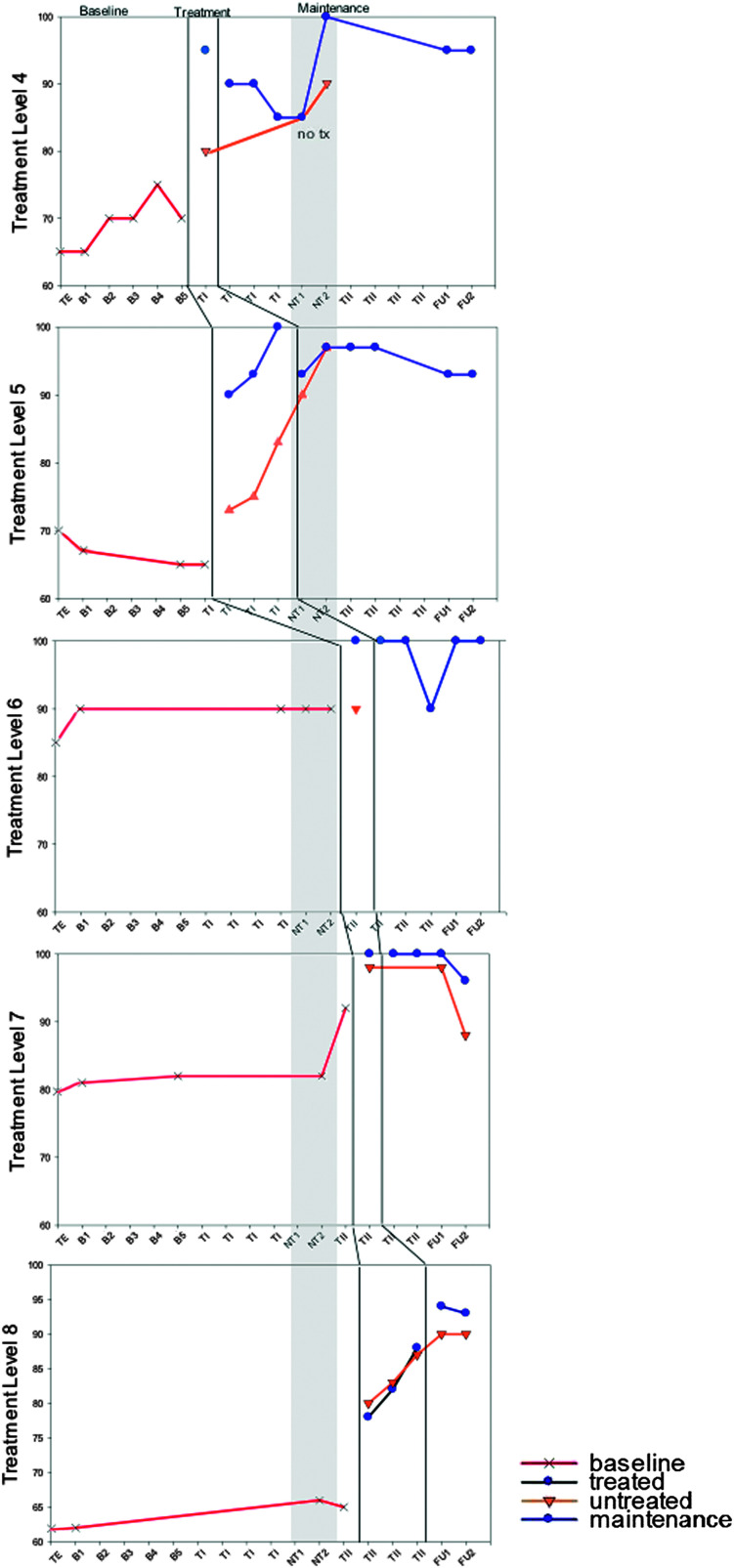
M1 accuracy (% correct) for Treatment Levels 4–8. TE = treatment level establishment; B = baseline; TI = Treatment Period I; NT = no-treatment period (the first NT time point occurred immediately post Treatment Period I; the second occurred immediately prior to Treatment Period II); TII = Treatment Period II; FU1 = immediate post treatment; FU2 = 8 weeks post treatment; tx = treatment. Note that activity between the vertical lines depicts treatment at the level defined. Activity beyond the vertical lines depicts maintenance of that treatment level when subsequent levels are the focus of treatment. Activity prior to the vertical line depicts performance prior to treatment at that level.

**Figure 3.**
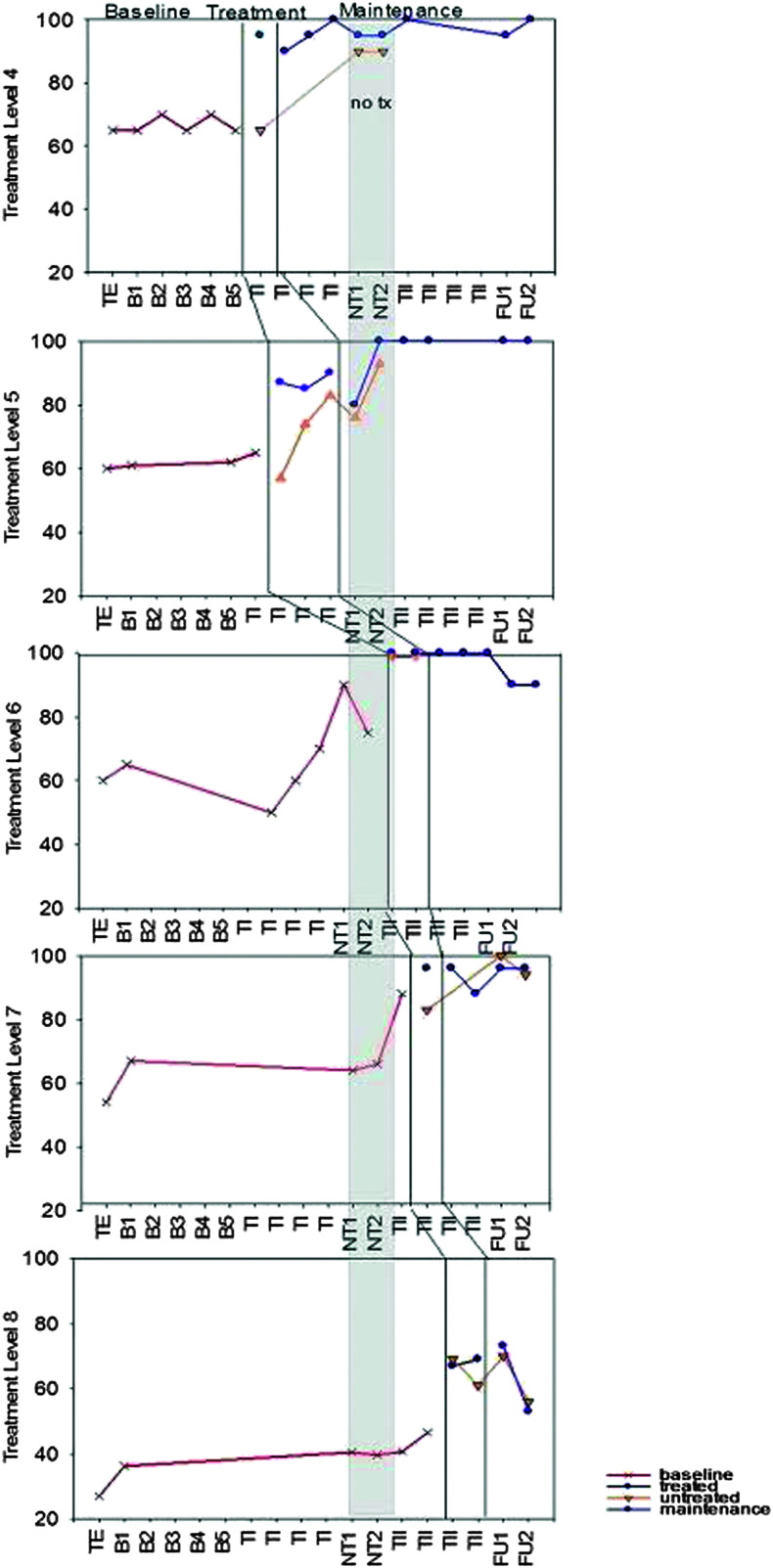
M2 accuracy (% correct) for Treatment Levels 4–8. TE = treatment level establishment; B = baseline; TI = Treatment Period I; NT = no-treatment period (the first NT time point occurred immediately post Treatment Period I; the second occurred immediately prior to Treatment Period II); TII = Treatment Period II; FU1 = immediate post treatment; FU2 = 8 weeks post treatment. Note that activity between the vertical lines depicts treatment at the level defined. Activity beyond the vertical lines depicts maintenance of that treatment level when subsequent levels are the focus of treatment. Activity prior to the vertical line depicts performance prior to treatment at that level.

**Figure 4.**
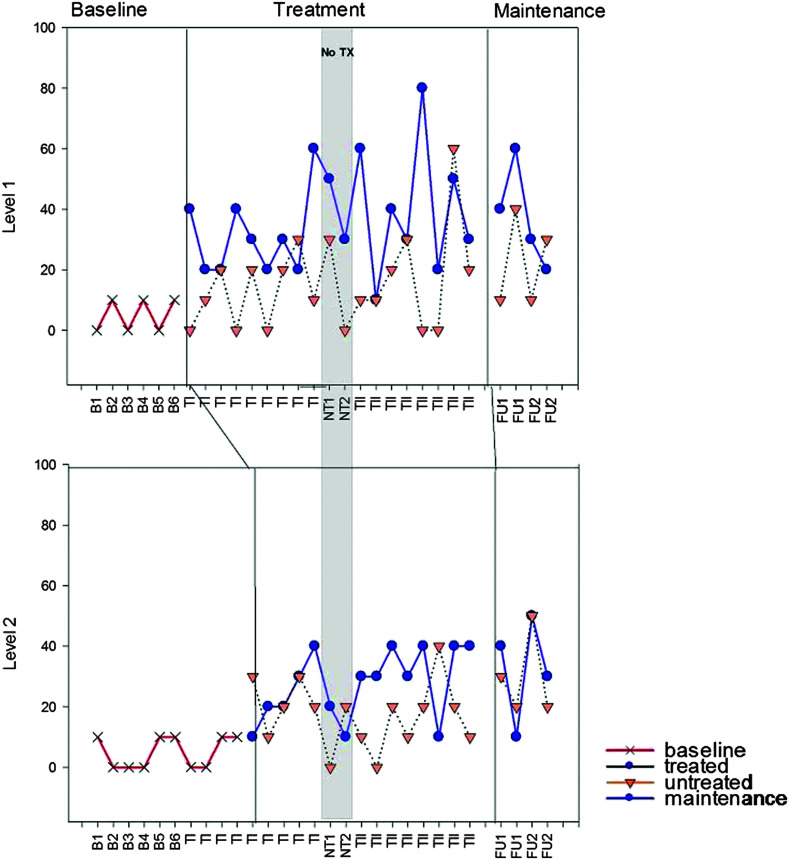
S1 accuracy (% correct) for Treatment Levels 1–2. B = baseline; TI = Treatment Period I; NT = no-treatment period (the first NT time point occurred immediately post Treatment Period I; the second occurred immediately prior to Treatment Period II); TII = Treatment Period II; FU1 = immediate post treatment; FU2 = 8 weeks post treatment. Note that activity between the vertical lines depicts treatment at the level defined. Activity beyond the vertical lines depicts maintenance of that treatment level. Activity prior to the vertical line depicts performance prior to treatment at that level.

**Figure 5.**
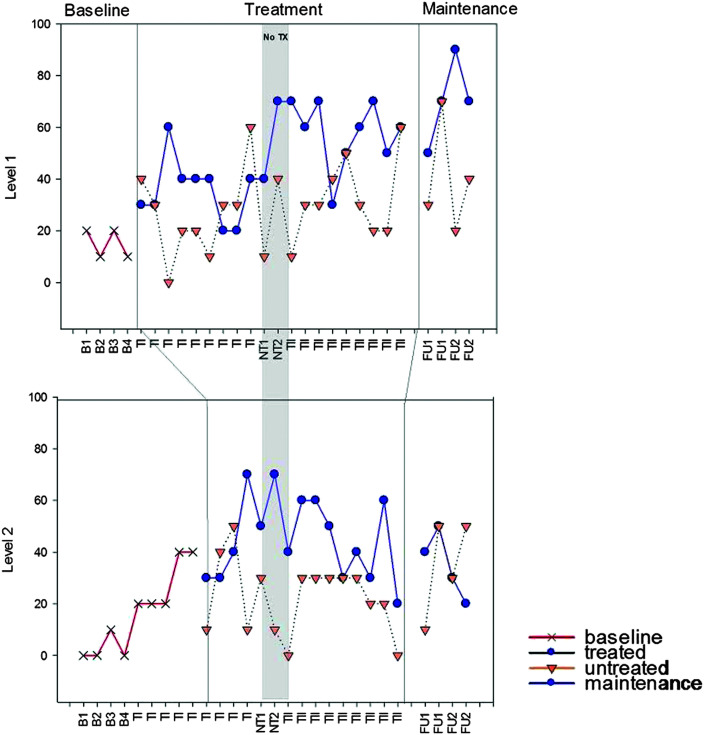
S2 accuracy (% correct) for Treatment Levels 1–2. B = baseline; TI = Treatment Period I; NT = no-treatment period (the first NT time point occurred immediately post Treatment Period I; the second occurred immediately prior to Treatment Period II); TII = Treatment Period II; FU1 = immediate post treatment; FU2 = 8 weeks post treatment. Note that activity between the vertical lines depicts treatment at the level defined. Activity beyond the vertical lines depicts maintenance of that treatment level. Activity prior to the vertical line depicts performance prior to treatment at that level.

**Figure 6.**
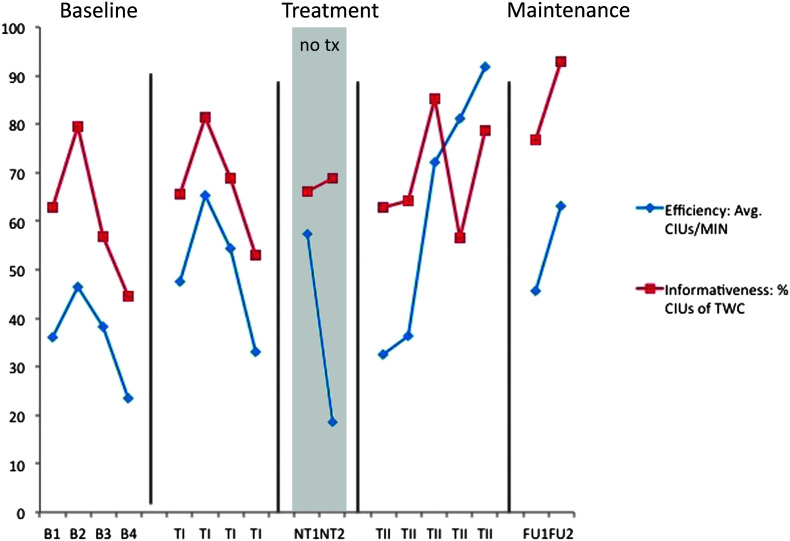
M1 narrative discourse probes—efficiency and productivity. B = baseline; TI = Treatment Period I; NT = no-treatment period (the first NT time point occurred immediately post Treatment Period I; the second occurred immediately prior to Treatment Period II); TII = Treatment Period II; FU1 = immediate post treatment; FU2 = 8 weeks post treatment; CIUs = correct information units; MIN = minute; TWC = total word count. Note that the *y*-axis represents both integer values (efficiency) and percent values (informativeness).

**Figure 7.**
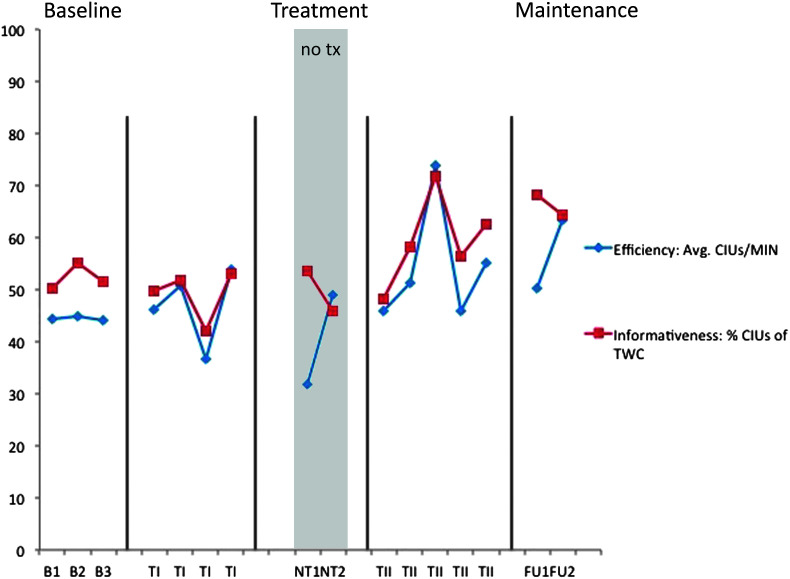
M2 narrative discourse probes—efficiency and productivity. B = baseline; TI = Treatment Period I; NT = no-treatment period (the first NT time point occurred immediately post Treatment Period I; the second occurred immediately prior to Treatment Period II); TII = Treatment Period II; FU1 = immediate post treatment; FU2 = 8 weeks post treatment; CIUs = correct information units; MIN = minute; TWC = total word count. Note that the *y*-axis represents both integer values (efficiency) and percent values (informativeness).

**Figure 8.**
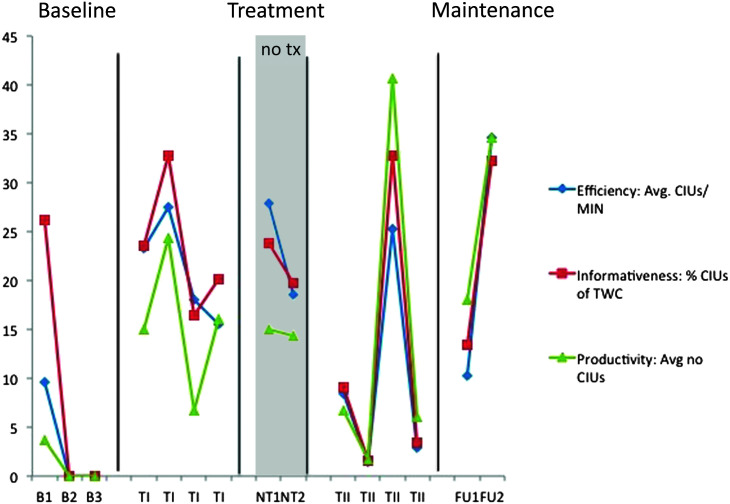
S1 narrative discourse probes—efficiency and productivity. B = baseline; TI = Treatment Period I; NT = no-treatment period (the first NT time point occurred immediately post Treatment Period I; the second occurred immediately prior to Treatment Period II); TII = Treatment Period II; FU1 = immediate post treatment; FU2 = 8 weeks post treatment; CIUs = correct information units; MIN = minute; TWC = total word count. Note that the *y*-axis represents both integer values (efficiency and productivity) and percent values (informativeness).

**Figure 9.**
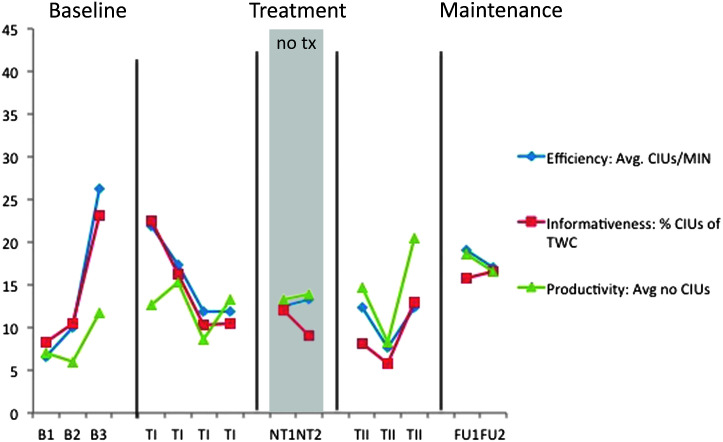
S2-narative discourse probes—efficiency and productivity. Note that the *y*-axis represents both integer values (efficiency and productivity) and percent values (informativeness). B = baseline; TI = Treatment Period I; NT = no-treatment period (the first NT time point occurred immediately post Treatment Period I; the second occurred immediately prior to Treatment Period II); TII = Treatment Period II; FU1 = immediate post treatment; FU2 = 8 weeks post treatment; CIUs = correct information units; MIN = minute; TWC = total word count.

Results from standardized assessment, probes of trained and untrained materials, and discourse performance are summarized below. Details of individual performance are contained within the figures and tables referenced in each section (Figures 2, 3, 4, 5, 6, 7, 8, and 9 and Tables 2 and 3), and a summary of the data from all participants is depicted in Table 4. Treatment probe results for all participants are summarized in multiple baseline formats representing percent accuracy (see Figures 2, 3, 4, and 5). Performance is depicted on trained and untrained materials over six phases shown on the *x*-axis for each individual following the probe used to determine level of treatment. Phases include TE, pretreatment baseline phase (B1–B5 for M1 and M3 and B1–B6 for S1 and S2), Treatment Period I (T1), no treatment (NT1-2), Treatment Period II (TII), immediate post treatment (FU1), and 8 weeks post treatment (FUII). For S1 and S2, TE and B1 were the same and are identified as B1 on the figures. Each figure includes the two probes that took place during the no-treatment period at each level. The first was taken in the week immediately following Treatment Period I, and the second was taken 4 weeks later just prior to the beginning of Treatment Period II. Note that figures for M1 and M2 show results for the four levels of treatment that they completed and the one they were still working on (Levels 4–8 marked on the *y*-axis), and S1 and S2 show results for the two levels of treatment to which they were exposed. Data points subsequent to treatment data are follow-up probes used to assess for maintenance following cessation of treatment at that level. Follow-up points occurred at all levels the week following treatment and, again, 8 weeks post treatment. Greater numbers of follow-up points tend to translate to better reliability of performance at a given time period. This is especially true for people with more severe aphasia who tend to demonstrate more variability in performance from trial to trial. Given the limited number of hours allotted for follow-up testing, it was not possible to collect more than two data points per level for M1 and M2. S1 and S2 only completed two levels, so it was possible to collect four data points for each.

**Table 2.** Assessment scores at each testing period.

M1									M2							
Assessment	Pre-Tx	Post-Tx I	% change for Tx I	Post-Tx II	% change for Tx II	% change both	F/U	% change maintained	Pre-Tx	Post-Tx I	% change for Tx I	Post-Tx II	% change for Tx II	% change both	F/U	% change maintained
			Pre-Tx–Tx I		Post-Tx I–Post-Tx II	Pre-Tx–Tx II		Pre-Tx–F/U			Pre-Tx–Tx I		Post-Tx I–Post-Tx II	Pre-Tx–Tx II		Pre-Tx–F/U
BNT	92.0	93.0	1.1	94.0	1.1	2.2	97.0	5.4	76.7	80.0	4.3	86.7	8.3	13.0	90.0	17.4
CRTT	91.3			94.0		2.9	91.7	0.4	72.0			83.3		15.7	81.3	13.0
WAB-R AQ	95.0	97.6	2.7	99.6	2.0	4.8	97.8	2.9	87.6	93.8	7.1	95.8	2.1	9.4	93.9	7.2
WAB-R CQ	95.2			98.7		3.7	98.6	3.6	90.3			94.5		4.7	94.7	4.9
WAB-R LQ	93.5			98.0		4.8	98.5	5.3	87.6			93.8		7.1	95.0	8.4

Subtests from the Western Aphasia Battery–Revised																

Spontaneous speech	95.0	95.0	0.0	100.0	5.3	5.3	97.5	2.6	95.0	95.0	0.0	100.0	5.3	5.3	97.5	2.6
Auditory verbal comprehension	100.0	100.0	0.0	100.0	0.0	0.0	100.0	0.0	91.0	96.0	5.5	96.0	0.0	5.5	85.5	−6.0
Repetition	96.0	100.0	4.2	100.0	0.0	4.2	100.0	4.2	69.0	86.0	24.6	87.0	1.2	26.1	90.0	30.4
Naming and word finding	89.0	98.0	10.1	98.0	0.0	10.1	94.0	5.6	88.0	97.0	10.2	96.0	−1.0	9.1	94.0	6.8
Object naming	93.3	100.0	7.2	100.0	0.0	7.2	100.0	7.2	96.7	100.0	3.4	100.0	0.0	3.4	100.0	3.4
Word fluency	65.0	90.0	38.5	90.0	0.0	38.5	70.0	7.7	50.0	85.0	70.0	80.0	−5.9	60.0	70.0	40.0
Sentence completion	100.0	100.0	0.0	100.0	0.0	0.0	100.0	0.0	100.0	100.0	0.0	100.0	0.0	0.0	100.0	0.0
Responsive speech	100.0	100.0	0.0	100.0	0.0	0.0	100.0	0.0	100.0	100.0	0.0	100.0	0.0	0.0	100.0	0.0
Reading score	100.0			100.0		0.0	100.0	0.0	96.0			96.0		0.0	100.0	4.2
Writing score	80.0			95.0		18.8	98.0	22.5	88.5			86.0		−2.8	98.0	10.7
Apraxia score	98.3			100.0		1.7	100.0	1.7	98.3			100.0		1.7	100.0	1.7
Constructional, visuospatial, and calculation scores	95.0			94.0		−1.1	99.0	4.2	89.0			89.0		0.0	95.0	6.7
RCPM	89.2			83.8		−6.1	97.3	9.1	86.5			94.6		9.4	89.2	3.1
**S1**									**S2**							
**Assessment**	**Pre-Tx**	**Post-Tx I**	**% change for Tx I**	**Post-Tx II**	**% change for Tx II**	**% change both**	**F/U**	**% change maintained**	**Pre-Tx**	**Post-Tx I**	**% change for Tx I**	**Post-Tx II**	**% change for Tx II**	**% change both**	**F/U**	**% change maintained**
			**Pre-Tx–Tx I**		**Post-Tx I–Post-Tx II**	**Pre-Tx I–Tx II**		**Pre-Tx I–F/U**			**Pre-Tx–Tx I**		**Post-Tx I–Post-Tx II**	**Pre-Tx I–Tx II**		**Pre-Tx I–F/U**
BNT	3.3	10.0	200.0	21.7	116.7	550.0	18.3	450.0	5.0	15.0	200.0	20.0	33.3	300.0	20.0	300.0
CRTT	76.7			83.3		8.7	81.3	6.1	76.0			70.0		−7.9	60.7	−20.2
WAB-R AQ	38.5	52.5	36.4	52.9	0.8	37.4	52.3	35.8	51.7	64.0	23.8	62.5	−2.3	20.9	64.4	24.6
WAB-R CQ	53.6			62.8		17.3	63.2	17.9	60.6			69.6		14.9	70.6	16.5
WAB-R LQ	43.5			54.7		25.6	54.8	26.0	52.6			65.5		24.5	68.6	30.4

Subtests from the Western Aphasia Battery–Revised																

Spontaneous speech	25.0	35.0	40.0	35.0	0.0	40.0	35.0	40.0	65.0	75.0	15.4	75.0	0.0	15.4	70.0	7.7
Auditory verbal comprehension	77.5	85.5	10.3	85.5	0.0	10.3	81.5	5.2	71.0	92.0	29.6	77.5	−15.8	9.2	83.0	16.9
Repetition	41.0	65.0	58.5	69.0	6.2	68.3	50.0	22.0	50.0	40.0	−20.0	33.0	−17.5	−34.0	39.0	−22.0
Naming and word finding	24.0	42.0	75.0	40.0	−4.8	66.7	40.0	66.7	28.0	48.0	71.4	52.0	8.3	85.7	60.0	114.3
Object naming	28.3	50.0	76.5	40.0	−20.0	41.2	50.0	76.5	33.3	43.3	30.0	58.3	34.6	75.0	63.3	90.0
Word fluency	0.0	0.0	0.0	5.0	n/c	n/c	15.0	n/c	5.0	20.0	300.0	30.0	50.0	500.0	15.0	200.0
Sentence completion	70.0	80.0	14.3	90.0	12.5	28.6	90.0	28.6	30.0	90.0	200.0	50.0	−44.4	66.7	100.0	233.3
Responsive speech	0.0	40.0	n/c	60.0	50.0	n/c	60.0	n/c	40.0	90.0	125.0	60.0	−33.3	50.0	90.0	125.0
Reading score	60.0			58.0		−3.3	65.0	8.3	44.0			65.5		48.9	79.5	80.7
Writing score	22.5			40.0		77.8	37.5	66.7	55.0			67.0		21.8	61.0	10.9
Apraxia score	90.0			90.0		0.0	90.0	0.0	90.0			91.6		1.8	90.0	0.0
Constructional, visuospatial, and calculation score	93.0			90.0		−3.2	96.0	3.2	68.0			82.0		20.6	77.0	13.2
RCPM	94.6			94.6		0.0	89.2	−5.7	48.6			70.3		44.4	64.9	33.3

*Note.* All scores shown as percent of the maximum score except for the WAB-R composite scores (AQ, CQ, LQ), which are shown in points. Blanks indicate that the test or subtest was not administered post Treatment Period I, and therefore, % change scores could not be calculated. Tx = treatment period; F/U = follow-up; BNT = Boston Naming Test; CRTT = Computerized Revised Token Test; WAB-R AQ = Western Aphasia Battery–Revised Aphasia Quotient; WAB-R CQ = Western Aphasia Battery–Revised Cortical Quotient; WAB-R LQ = Western Aphasia Battery–Revised Language Quotient; RCPM = Raven's Coloured Progressive Matrices; n/c = not calculable. Percent change = ((y2 – y1) / y1) × 100.

**Table 3.** Effect sizes.

Participant	Probe	Effect	Treatment Period I effect	Treatment Period II effect	Total treatment effect	Follow-up effect
		Description	Pre-Tx–immediate Post-Tx I	Post-Tx I–immediate Post-Tx II	Pre-Tx I–immediate Post-Tx II	Pre-Tx I–8 week follow-up
M1	Treatment	Level 5-trained	**8.51**	N/C	**8.54**	**8.54**
		Level 5-untrained	**7.47**	−0.18	**7.47**	**7.47**
		Level 8-trained	1.67	**40.31**	**15.95**	**15.48**
		Level 8-untrained	1.67	**31.82**	**12.62**	**14.05**
	Discourse	Average CIUs	0.67	0.23	0.26	−0.40
		CIUs/MIN	3.13	0.28	1.63	3.85
		CIUs:TWC	0.35	**4.66**	1.12	2.29
M2	Treatment	Level 5-trained	**27.58**	0.00	**56.43**	**56.43**
		Level 5-untrained	**21.92**	0.71	**45.96**	**13.00**
		Level 8-trained	**5.66**	**8.31**	**21.19**	**11.67**
		Level 8-untrained	**5.66**	**47.06**	**19.56**	**12.96**
	Discourse	Average CIUs	0.57	−0.63	−1.02	−0.07
		CIUs/MIN	**−32.09**	0.80	**14.79**	**48.09**
		CIUs:TWC	0.59	3.31	**6.60**	**4.93**
S1	Treatment	Level 1-trained	**8.22**	0.71	**8.18**	**3.64**
		Level 1-untrained	2.74	1.83	**8.18**	**6.36**
		Level 2-trained	**4.56**	1.83	**3.65**	2.74
		Level 2-untrained	−0.91	2.73	1.41	**5.45**
	Discourse	Average CIUs	**9.55**	**7.07**	**11.55**	**22.66**
		CIUs/MIN	**10.58**	−1.94	**3.52**	**13.27**
		CIUs:TWC	2.38	−2.90	1.07	3.42
S2	Treatment	Level 1-trained	**4.33**	0.24	**7.79**	**11.26**
		Level 1-untrained	−0.86	1.18	**6.03**	3.45
		Level 2-trained	**9.50**	−1.06	**8.50**	**4.50**
		Level 2-untrained	0.84	0.71	0.84	1.47
	Discourse	Average CIUs	2.09	**12.22**	**4.06**	3.26
		CIUs/min	−0.08	**12.17**	0.65	0.43
		CIUs:TWC	−0.19	2.51	0.37	0.48

*Note.* Effect sizes denoted in bold print are those that exceed the value for a small effect using benchmarks according to Beeson and Robey (2006): small (4), medium (7), large (10.1). Level 1 was trained during both treatment periods. Level 2 was trained for the second week of Treatment Period I and for all of Treatment Period II. Level 5 was only trained during Treatment Period I. Level 8 was only trained during Treatment Period II. Tx = treatment period; N/C = not calculable because no baseline variance; CIUs = correct information units; TWC = total word count.

**Table 4.** Score sheet denoting clinically significant changes across participants on five outcome measures.

Outcome measure	Probes												Standardized tests							
	Trained items				Untrained equivalent items				Narrative discourse				WAB-R AQ				BNT			
Indicator of clinical change					Effect size ≥ 4								≥ 5 points				≥ 20%			
Treatment period	Tx I	Tx II	Both	Maint	Tx I	Tx II	Both	Maint	Tx I	Tx II	Both	Maint	Tx I	Tx II	Both	Maint	Tx I	Tx II	Both	Maint
M1	√	√	√	√	√	√	√	√	—	√	—	*	—	—	5	—	—	—	—	—
M2	√	√	√	√	√	√	√	√	—	—	√	√	6	—	8	6	—	—	13	17
S1	√	—	√	*	—	—	√	√	√	√	√	√	14	—	14	14	200	117	550	450
S2	√	—	√	√	—	—	√	*	—	√	√	*	12	—	13	15	200	33	300	300

*Note.* Tx. I refers to the change from pretreatment to post Treatment Period I. Tx. II refers to change from post Treatment Period I to post Treatment Period II; both refers to pretreatment to post Treatment Period II. Maintenance refers to pretreatment to the 8-week post Treatment II follow-up. A checkmark within each treatment period denotes a clinical significance following that period according to the indicator of change for that outcome measure. An asterisk means the gain was approaching clinical significance. Em dashes indicate no clinically significant change. Actual values are provided for standardized scores. These have been rounded to the nearest whole number. Narrative discourse refers to the discourse outcome measure of interest for the participant (efficiency and informativeness for M1 and M2 and productivity for S1 and S2). WAB-R AQ = Western Aphasia Battery–Revised Aphasia Quotient; BNT = Boston Naming Test; Maint = Maintenance.

Effect sizes were calculated for two treatment levels per dyad and for the discourse production probes. This is calculated by subtracting the mean of the baseline points from the mean of the follow-up data points and dividing that by the standard deviation of the baseline. They were calculated from baseline to post–Treatment Period I to assess the Treatment Period I effect, from post–Treatment Period I to post–Treatment Period II to assess the Treatment Period II effect, from pre–Treatment Period I to post–Treatment Period II to assess the total treatment effect, and from pre–Treatment Period I compared with the 8-week follow-up point to determine whether there was a follow-up effect and if gains were maintained. This is depicted in Figure 1. All effect sizes were calculated based on two to six baseline observations and one to four follow-up data points depending on the treatment level. More pretreatment baselines points were available for the starting levels of treatment, for example.

It is important to interpret effect sizes cautiously and in conjunction with the visual inspection of individual figures because baseline variability, or lack thereof, influences the quotient. The more constant the baseline, the more likely any increase appears to be attributed to treatment. In some cases, visual inspection shows a pattern of slight variability and, thus, low effect sizes, despite the fact that posttreatment scores are well above baseline. In others, the baseline is so stable that the smallest improvement may be captured as having a clinically significant effect. Thus, there remains a need for visual inspection to aid interpretation.

Finally, a summative score sheet is provided as a way to compare across participants the clinically significant gains made following each treatment period for each participant on five different outcome measures (see Table 4).

### Standardized Assessment

Results are reported for performance on standardized measures as percent change following each treatment period, following both, and for maintenance of gains, if they were made. M1 and M2 made small, nonclinically significant gains following each treatment period. Changes following both treatment periods together were larger for both participants and were clinically significant on the WAB-R AQ and the BNT for M2. S1 and S2 made large gains (both > 13 points or > 23.4%) on the WAB-R AQ following Treatment Period 1 only. On the BNT, however, participants continued to show clinically significant gains following the second treatment period. Gains on the WAB-R AQ were observed across various subtests for each individual as depicted in Table 2. Little or no change was seen on the CRTT for M1 and S1. M2 increased by 13%, and S2 decreased with each administration. See Table 2 for scores for each measure at each time period.

### Probes of Trained and Untrained Stimuli

In order to analyze change over time, each participant's probe results for trained and untrained stimuli are depicted individually in Figures 2, 3, 4, and 5. Results are shown for each of the dependent measures, including percent accuracy at each level of treatment for trained and untrained materials, and productivity, efficiency, and informativeness of connected speech from the discourse production samples. All participants attended all baseline, assessment, and follow-up probe sessions as scheduled, and all completed all 60 hr of treatment, except for S1 who missed 1 day and, thus, completed 57 of the 60 hr of treatment. As previously stated, M1 and M2 received two follow-up probes per level, whereas S1 and S2 received four per level. M1 and M2 completed five treatment levels and had to complete a total of 10 follow-up probes. S1 and S2 completed two treatment levels and, so, completed a total of eight follow-up probes.

M1 and M2 began the first treatment administration at Level 4 but spent the majority of this time period at Level 5, which was mastered with criterion of 80% accuracy by both participants. After 5 weeks of no treatment, they started Treatment Period II at Level 6 but mastered it and Level 7 quickly and spent the majority of this period on Level 8. M1 was the first to achieve criterion of 80% accuracy at all levels and mastered Level 8 by the end. M2 had not yet reached criterion for Level 8 by the end of the second treatment period (see Figures 2 and 3).

Both participants demonstrated medium to large effects for each treatment period for both trained and untrained stimuli (see Table 3). Note that lack of an additional effect at Level 5 following Treatment Period II is consistent with the fact that these stimuli were no longer part of the training materials. A negative effect would have been expected only if mastered performance had not been maintained.

S1 and S2 started at Level 1 and were introduced to Level 2 one week later. The second treatment administration ended at Level 2 prior to mastery for both participants (see Figures 4 and 5). Each achieved medium to small effects, respectively, for trained materials only following Treatment Period I but no additional significant effects were observed following Treatment Period II (see Table 3). Slightly larger effects were observed following both treatment periods (total treatment effect, see Figure 1), including small effects for untrained Level 1 items for both participants. Effects were generally maintained at follow-up for all four participants.

### Generalization Probes of Narrative Discourse

The individual results of the discourse production probes are shown for participants in Figures 6, 7, 8, and 9. We were particularly interested in productivity for S1 and S2 and in efficiency and informativeness of discourse for M1 and M2. Productivity was measured as the number of CIUs per sample. Three samples were provided per probe, and an average CIU count was calculated. Because pretreatment discourse productivity was judged as generally appropriate for M1 and M2, it was not considered a measure of interest, though it was analyzed in the unlikely event of change. Given that there was no trend of increase or decrease over time for either participant, this measure is not clinically of interest and, therefore, is not depicted here. For discourse efficiency, the number of CIUs per minute was calculated, and informativeness was the proportion of CIUs to TWC (Nicholas & Brookshire, 1993), shown as a percentage. Efficiency and informativeness are depicted for all four participants.

For M1, variability was observed for both efficiency and informativeness performance as seen in Figure 6. No change was observed for productivity, as anticipated (average of 25–50 CIUs per session, not depicted). A steep, rising slope in CIUs per minute is observable within Treatment Period II, but this did not correspond with a clinically significant effect for this measure of efficiency. There was, however, a small Treatment Period II effect for informativeness (CIUs as a percent of TWC). A Follow-up effect is apparent with visual inspection only.

No change was expected or observed in productivity for M2 (average of 150–200 CIUs per probe session, not depicted). This participant had a more stable baseline for CIUs per minute than for CIUs as a percent of TWC; therefore, calculations of efficiency yielded a large negative Treatment Period I effect and large positive total treatment and follow-up effects on this measure. No Treatment Period II effect was observed. Positive treatment effects were not observed following Treatment Period I or Treatment Period II for discourse informativeness, but there were small total treatment and follow-up effects for this measure. Again, using visual inspection to inform these results, in this case, it is clear that there was an upward trend for both measures despite variable performance throughout. For both measures, a small rise in slope was evident throughout and following Treatment Period I followed by a moderate increase in slope within and following Treatment Period II.

Productivity was the primary discourse measure for S1 and S2. Efficiency and informativeness measures have been included to provide consistency between participants, but because the production of informational content was so compromised in both of these participants, productivity was the outcome variable of interest. For S1, medium Treatment Period I and Treatment Period II effect sizes and large total treatment and follow-up effects were calculated for productivity (see Table 3 and Figure 8). Variability in S1's productivity was attributed to his use of overlearned phrases. He was often able to use his limited repertoire in such a way that they were appropriate to the picture and could be counted as CIUs. A large Treatment Period I effect, a small total treatment effect, and large follow-up effect were observed for efficiency. No treatment effect was observed for informativeness.

S2's productivity increased as observed in Figure 9 with a large Treatment Period II effect and a small total treatment effect. She showed a large Treatment Period II effect for efficiency and no effect for other periods or for the measure of informativeness.

## Discussion

This study investigated the treatment response of four people with chronic aphasia, each provided with two rounds of CILT, administered for 3 hr per day over 2 weeks and separated by a 5-week no-treatment period. Each participant was more than 2.5 years post onset at the time of treatment initiation, well past the point of spontaneous recovery. It was predicted that an additional intensive treatment administration (30 hr over 2 weeks) would result in progression through additional treatment levels and additional gains on standardized tests and on other outcome measures. Two of the four participants had mild aphasia for which the application of CILT is relatively unsupported. It was predicted that, by making CILT sufficiently challenging through the use of increasingly difficult stimuli decks, gains would be observed. Further, we predicted that any gains were more likely to be captured on the generalization measure of discourse performance than on standardized assessment for this population, given that their scores were near ceiling for standardized measures. Considering that, in previous studies, generalization has been shown following a 2-week period (Maher et al., 2006), it was anticipated that generalization effects would be greater with the additional treatment period. Finally, we predicted that language gains would be maintained for some, if not all, participants, again given the positive results following only 2 weeks of CILT in multiple studies (Barthel et al., 2008; Maher et al., 2006; Rose et al., 2013). Though often overlooked in treatment studies, durability of treatment gains is a critical component in assessing a program for clinical practice. It is possible that, even if an additional treatment administration was not successful in conferring additional language advantage, it may still contribute to long-term maintenance.

### Responsiveness to CILT After One and Two Treatment Administrations

A single administration of CILT resulted in language gains similar to those seen in previous studies that utilized CILT for approximately 30 hr over 2 weeks (e.g., Breier et al., 2006; Meinzer et al., 2005). Comparable to previous studies, three of the four participants improved on the WAB-R AQ by at least 5 points and two of the four on the BNT. Two showed generalization to untrained materials, and one showed a clinically significant effect of treatment on discourse (see summative score sheet, Table 4). Treatment Period I was shown to be clinically effective for all participants on at least two outcome measures, providing confirmation that CILT provided at 30 hr per week over 10 consecutive working days can be beneficial to individuals with chronic aphasia. It was predicted that a second administration of treatment would result in increased accuracy of productions, increases on standardized tests, and increases on generalization measures. Instead, only the two participants with mild aphasia increased in accuracy to both trained and untrained items and only S1 and S2 made additional clinically significant changes on the BNT. Additional gains were not achieved on the WAB-R AQ for any participant.

The goal of any treatment is the ultimate use of trained skills in everyday life, extending beyond the materials from the clinic, yet measures of generalization are not often included in treatment studies. Three of the four participants showed improvements in discourse, and all four participants maintained their gains from the first and second treatment periods at the 8-week follow-up. When the outcome data are viewed together for all participants, it appears that the second treatment was of value, although arguably of lesser value than the first treatment period. Again, each participant demonstrated clinically significant change on at least two measures.

Of interest is the increase in performance on the writing subtest of the WAB-R for all participants and on the RCPM for three of the four participants. In the case of the M1 and M2, maximum changes were observed at the follow-up testing, though all four participants showed changes following Treatment Period II. Writing subtests all improved by at least 10 percentage points, and RCPM scores increased by 9–12 points for three of the four participants. These were unanticipated gains, not previously reported in studies using CILT, and it remains unclear as to why there were improvements in two modalities that were never trained and as to how much the second treatment administration contributed to changes in these areas. There are at least two possibilities for these results: (a) The participants may be getting better at performing on these measures as they see each three to four times within 4.5 months. This is especially true for the participants with mild aphasia who are performing at ceiling and may take note of specific words or areas of difficulty to practice on their own. On the other hand, noting incorrect instances of reading and writing or pattern discernment (on the RCPM) seems less plausible than noting difficulty with a particular word production. (b) Another possibility is that treatment had far-reaching effects and contributed to gains in other communicative modalities and in cognition more than anticipated.

Other changes were evident that were not captured in testing. For example, S1 and S2 both became more responsive to cueing, and S1 decreased his use of an entrenched stereotypy. Where it was used previously as filler, S1 appeared to become more comfortable with silently working toward finding a word. Video review showed a 90% decrease in the use of the stereotypy from before treatment to follow-up. Unsolicited, positive feedback was received from family members of all four participants. Sensitive quality-of-life measures that can be repeated multiple times are needed to capture these kinds of changes over a short time.

### Influence of Severity on Outcomes

The two participants with mild aphasia made greater improvements on both the trained and untrained items and achieved mastery on several treatment levels compared with the two with more severe aphasia who never achieved 80% accuracy at any level. Generalization to untrained items, stimuli to which the participants had never been exposed, occurred beyond what was predicted for M1 and M2 and resulted in large effect sizes that were nearly equivalent to those seen on trained items. This occurred either simultaneously or closely following the point at which criterion was reached.

It is important to recall that M1 and M2 were exposed to hundreds of trained items for each level of treatment. This approach was based on the rationale that the training goal was to stimulate language processes rather than to memorize lists of words. Reengaging disrupted language processes should be the goal of any therapy regimen targeting oral–verbal language production. With the return of spontaneous language, it is hoped that a cascade effect is initiated, freeing up cognitive resources for another aspect of language function. For example, in this case, once accurate and complex sentences are produced reliably, the next natural step might be to become more fluent or more efficient in the delivery of productions.

More modest treatment gains were observed for S1 and S2 with less generalization to untrained words and small to medium effect sizes demonstrated only after Treatment Period II. For all participants, follow-up probes of untrained materials exceeded those recorded pretreatment at baseline. Despite this, greater gains on standardized tests were achieved for these two participants than for M1 and M2, indicating a clinically significant change in aphasia severity.

Generalization to discourse was anticipated for all. Efficiency and informativeness were expected to improve for M1 and M2 for whom productivity was not judged to be a problem, whereas productivity was the main outcome measure for S1 and S2. Effect sizes on these measures varied between participants and following each treatment session, but visual inspection revealed an upward trend for all four participants. The trend is clearer if one considers average pretreatment values compared with the average of those at follow-up. Although discourse performance was variable from day to day, lowest performances at 2 months post treatment still exceeded high performances observed prior to treatment (see Figures 6 and 7 to observe this for efficiency and informativeness and Figures 8 and 9 for productivity).

In sum, it appears that those with mild aphasia improved directly on the specific tasks being trained, and this generalized to untrained tasks. Those with more severe aphasia did not demonstrate the same robust treatment effect and yet made clinically significant gains on standardized batteries.

### Durability of Treatment Effects

It was predicted that gains in response accuracy for trained material and untrained materials and gains on standardized test scores and in discourse productivity, efficiency, and informativeness would all be maintained 8 weeks post Treatment Period II.

Durability of treatment effects is an encouraging outcome that has been observed following intensive treatment protocols such as this. When Maher et al. (2006) contrasted two equally intensive treatments, she noted that those who participated in CILT tended to maintain language gains better than those who participated in a multimodality treatment. Animal studies have shown that physiologic changes to the injured motor cortex may require hundreds of repetitions (Nudo & Milliken, 1996), and musician studies show that practice is necessary to maintain neural change (Pascual-Leone, Amedi, Fregni, & Merabet, 2005). Once again, translation to the reestablishment of language in the injured brain is less straightforward; however, it follows that increased accurate oral–verbal productions will result in neural and behavioral change. When the change is increased verbal output, practice can continue organically and is more likely to be maintained.

In the current study, maintenance was observed on nearly all measures, including primary measures of performance on trained exemplars, secondary measures of standardized tests, and measures of generalization. Change on some subtests for some participants were maximized following Treatment Period I, some following Treatment Period II. On subtests where a decrease was noted between these two time periods, it tended to be recouped at the follow-up assessment, such that nearly all gains made at either treatment period were maintained post treatment. Performance on the repetition subtest was one exception for both S1 and S2. S1 made incremental progress on this subtest over each treatment period, but follow-up scores dipped down close to baseline. S2 decreased in repetition proficiency during each treatment period and appeared to regain some at follow-up, though not back to baseline levels. It is not clear how treatment might have influenced negative change in this one area, but it is possible that the injured system is competing for limited resources and impacting processes that have not been the subject of focus.

Durability of treatment effects has not always been observed following CILT. In response to a participant's drop in language gain 7 months post treatment, Kurland et al. (2012, p. S82) postulated that, perhaps, an intensive short-term treatment “provides a spark, but not continuous fuel, for ongoing recovery in some individuals.” Perhaps, a more powerful spark is necessary to generate that continuous fuel in some individuals. Although the current treatment results cannot yet be generalized to others with aphasia, these data are promising, and it would be useful to test the double treatment administration on a larger sample, with a longer follow-up period, to determine whether 60 hr of treatment might be the more powerful spark needed to ensure maintenance of gains.

### The Role of CILT

Opponents of CILT tend to take issue with constraint to the verbal modality. Disallowing any form of expressive communication in an individual with aphasia runs contrary to the clinical mindset and to studies demonstrating that gesture may facilitate language production. In reality, CILT's emphasis on the verbal modality is no different from any other treatment of oral–verbal expression. Response elaboration therapy (Kearns, 1985) and semantic features analysis (Boyle & Coelho, 1995) are two examples of treatments in which oral–verbal language is produced repeatedly in order to improve this specific modality. Both of these treatments have been demonstrated to be effective and, if contrasted, might be predicted to produce superior results to CILT as each would be tailored to an individual's needs and also because these treatment types take place individually, allowing more opportunity for repetition and, thus, potential for neural change. CILT's group design is more focused on productive interactions and is not customized to the individual. Despite this, no other treatment type is reported as having the consistent positive changes in pre–post standardized test scores, overall generalizability, and maintenance of gains seen following CILT. This may be due to the fact that these alternate treatments are rarely administered at the same consistently high intensity.

The group aspect of CILT warrants careful consideration. The potential drawback of shared time for verbal productions and fewer teaching episodes may be outweighed by the positive effects, one of which might include peers working together. The card game, central to CILT, is repetitive, and maintaining focus for 3 hr was expected to be challenging. No decrease in interest was observed, however, likely due to the competitive nature of the game. The support and encouragement from others with aphasia may also be valuable. Semantic features analysis tends to be administered in individual treatment, but recent group trials have shown promising results (Antonucci, 2009) supporting the group format as another variable that may contribute to outcomes seen following CILT. Group work and intensity appear to play significant roles; however, in the few studies that have administered an alternative treatment and CILT at equal intensities and both in group settings, some find at least slight advantage with CILT. Maher et al. (2006) reported better maintenance, Kurland et al. (2012) reported better naming, and Rose et al. (2013) reported no differences. The role of intensity and group effect of various treatments should continue to be empirically tested, but in the meantime, CILT remains an effective tool for both treatment and research purposes, offering consistently positive outcomes for participants.

### Limitations of the Study

This study was multifaceted and complicated with several outcome measures adapted for use with a heterogeneous patient population of varying aphasia severity. As a result, several limitations should be taken into consideration while interpreting the results.

#### Progress in Naming Was Not Adequately Captured for S1 and S2

Tracking success at a lower starting level (accuracy when provided a phonemic or semantic cue) would have provided more information about progress for these participants. The tracking of cues only began when it was clear S1 was progressing with cues even when he did not appear to be progressing with spontaneous naming. By the end of Treatment Period II, a single initial phonemic cue resulted in 100% accuracy for trained words and 80% accuracy for untrained words. This was increased from 20% on each when documentation of cueing began on Week 2 of Treatment Period I.

#### Generalization to Subsequent Treatment Levels Weakens Design

Regular increases in subsequent levels showed generalization of treatment for M1 and M2, which was a positive outcome for participants but calls into question the experimental control of the design. This is a common problem within treatment research, as anytime a patient exceeds expectations, the methods must be revisited. Replication studies and tighter experimental control are warranted but are unlikely to alleviate the problem altogether.

#### Probe Administration Should Be Performed by an Investigator Not Involved in Treatment

The first author performed all treatment and probe administration, which introduces the potential for bias in the recording of results.

Levels of difficulty were not individually based, and harder levels may not have been that much more challenging for one individual. Level 6, which required use of prepositions, for example, was considered a harder skill to perform consistently than noun and adjective production given literature reporting on substitution errors with prepositions even in anomia (Mätzig, Druks, Masterson, & Vigliocco, 2009). However, it did not appear to be more difficult for M1. This is a challenge of group design because materials cannot be customized for an individual.

#### More Data Following the No-Treatment Period Should Have Been Collected to Better Inform Responsiveness to the No-Treatment Period

Treatment probes collected immediately following Treatment Period I and prior to Treatment Period II provide data showing that gains were generally maintained. It is not necessarily clear whether standardized test scores also were maintained over this time period. If also administered prior to Treatment Period II, it would be possible to assess the impact of the no-treatment period and provide a better starting point by which to assess the change that occurred during Treatment Period II. This would mean five administrations of the same test materials. By using participants of like severity, perhaps fewer materials could be used, but it would remain an extensive amount of testing for the participants. It remains unclear as to whether a break is important for new neural processes to become fully instantiated or whether time off just allows recent changes to decay.

#### Repeated Measures May Lead to a Practice Effect

Despite good test–retest reliability on the standardized tests used, it is not standard practice to administer the same test four times within less than 6 months. A positive response due to practice effect is possible, particularly for those with milder deficits, and may account for the increases in scores on reading and writing subtests for these participants. Alternative outcome measures would benefit our field and help alleviate the problem of a potential practice effect.

#### More Homogeneity Between Participants Would Increase Interpretability of the Data

Four participants of similar severity would have strengthened findings. Outcome measures that were used for the participants with mild aphasia were not necessarily appropriate or achievable for those with moderate to severe aphasia. The CRTT is one example that elicited frustration for the latter group. The WAB-R AQ, in contrast, was too easy and not sensitive enough for the participants with mild aphasia.

## Conclusions and Future Directions

Evidence from this study suggests that a double treatment administration of CILT confers advantages over the single administration, but an optimal schedule remains unclear. Thirty hours, provided daily (session frequency), in 3-hr increments (session duration) and over 2 weeks (total intervention duration), appears to be an effective combination of treatment parameters as positive results are the consistent result of administration of CILT and other intensive therapies. Of particular interest, benefits do extend beyond simply increasing accuracy on treated tokens. Increasing the total intervention duration while keeping other intensity parameters constant has shown some benefit when a different language intervention was provided immediately after the first (Kurland et al., 2012; Rose et al., 2013). In the current study, when a second session of CILT was administered 5 weeks following completion of the first, there were gains noted in primary and at least one secondary outcome measure, although, in general, these gains were more modest than those observed after the first CILT administration. Importantly, gains were observed for all four participants despite the wide range of severity, including participants who tested at the mild end of the aphasia spectrum.

Individuals with mild aphasia tend not to be the focus of treatment studies and may be discharged from services prematurely due to their functional communication. This is unfortunate because this may be the group with the greatest likelihood of returning to employment and premorbid avocations. Correlations of initial aphasia severity and improvements on test scores have indicated that this population is less likely to benefit from CILT (Meinzer, Elbert, Djundja, Taub, & Rockstroh, 2007). Higher initial scores on standardized tests, however, may limit the sensitivity of these measures for detecting change secondary to language treatment. The current results strongly suggest that, given sufficiently challenging treatment stimuli, individuals with mild aphasia can show clinically significant gains on a number of treated and untreated measures after CILT.

## Appendix A

### Daily Probes of Treatment Items and Discourse

***Treatment Probes***

Treatment probes were administered daily to all participants prior to that day's treatment session. Probes included 20 trained and 20 untrained from the current treated items, 20 items from the subsequent level of treatment, and 20 from each of the completed levels to assess for maintenance of gains. Probes of subsequent levels also served as a continued baseline for that level until it was treated. Because M1 and M2 moved so quickly through the materials resulting in several completed levels to be probed, the process was split between 2 days, such that a complete set of probes was completed after every 6 hr of treatment.

For the participants with more severe aphasia (S1 and S2), all probes could be completed after every 3 hr because they never progressed beyond the single word level and, therefore, had no maintenance probes to complete. Probes for all persons with aphasia were always administered prior to treatment initiation, delivered via E-prime, and identical to the delivery that occurred during baseline testing.

Untrained materials were matched to trained materials by level. For example, in Level 5, two adjectives were required to differentiate among other items in the deck, so one trained deck might include several pans varying in color, type (frying pan vs. dust pan vs. child's toy pan), material (cast iron vs. plastic vs. ceramic), or number. The untrained deck would have different items, such as boots that also required two adjectives (women's dress boots vs. men's work boots or kids' ski boots). Nouns were controlled such that there were no duplicates between trained and untrained decks, but adjectives were not controlled, and colors and numbers were likely to be repeated.

Response accuracy was scored for all participants (see scoring details in Appendix B). No time limit was given for responses, and self-corrections were counted as accurate. No feedback was provided during the administration of probes. Participants were encouraged between probes, however, regardless of performance. For example, a persistent effort might elicit, “You're doing great!” or, if frustration was evident, “That's okay! Keep pushing yourself!”

Participants would see an untrained stimulus item a maximum of twice throughout the treatment study period (see Appendix B for information on stimuli used as probes). This was done in order to reduce the chance of improvement due to word exposure. Word frequency data were derived from the Medical Research Council (MRC) psycholinguistic database (Coltheart, 1981). This was relevant for training only for S1 and S2 who were at initial treatment levels requiring a separation of high and low frequency types.

Accuracy of production was calculated according to the scale outlined in Appendix B. Points earned were divided by the total possible points in order to determine percent accuracy at each level. In Levels 1 and 2, the total possible points were 1 per item for a total of 10 points. One wrong would result in a score of 80%. In Level 4 (word + adjective), the total possible points were 2 per item for a total of 20 points. Level 8 responses (one-sentence picture description) were scored for accuracy in a slightly different way. Instead of counting elements to be included, the percent accuracy of the utterance was recorded. To do this, probes were transcribed and scored for correct information units (CIUs) according to guidelines by Nicholas and Brookshire (1993) with one exception. Here, the word *and* was counted as a CIU in order that 100% accuracy in efficiency could be achieved. These were used to calculate the proportion of CIUs to total words in order to measure informativeness of oral–verbal production. Repeated or inaccurate words would detract from a total possible score of 100%. The production “The boy is preparing for a sock for the doctor” would count as eight CIUs out of a total 10 words so it would count as 80% accurate. (The boy is preparing for a shot from the doctor.)

***Discourse Probes***

Probes for generalization to narrative discourse were administered, before treatment sessions, on alternate days starting 9-hr posttreatment and, then, every 6 hr after that and were identical to the baselined discourse probes. In each probe, three of 12 Norman Rockwell pictures were presented with the request to “tell me what is happening in this picture.” Three new pictures were used in each future probe until all 12 had been used at which time the first three were shown again. Story descriptions were transcribed and scored for CIUs according to guidelines by Nicholas and Brookshire (1993). All picture descriptions were videotaped, transcribed verbatim, and analyzed for total CIUs to measure discourse productivity and were also used to calculate discourse efficiency (CIUs per minute) and discourse informativeness (the proportion of CIUs to total words). To account for potential differences in how much language each illustration has the potential to elicit, each of these measures were averaged across the three stimuli items resulting in a single data point.

## Appendix B

### Treatment Stimuli and Treatment Levels

***Treatment Stimuli***

Treatment stimuli consisted of 120 full-color stimulus items for each of eight levels for a total of 960 items. An additional 120 items per level were created that were never included in the training process and seen only 10 at a time during the treatment probe sessions. Items included on the Boston Naming Test and within the naming section of the Western Aphasia Battery–Revised were not included in the sets of stimuli in order to avoid inadvertent training for testing. This large number of stimulus items used is more than what tends to be reported in aphasia treatments, but it has been demonstrated that people with naming impairments of varying severity can tolerate and may benefit from much larger sets (Snell, Sage, & Ralph, 2010).

**Table:** Treatment hierarchy and scoring for treatment probes.

Level	Expected production	Max points/stimulus item	Total possible points
1	High-frequency object (1)	1	20
2	Low frequency object (1)	1	20
3	Mixed frequency object (1) + carrier phrase (1)	2	40
4	Mixed frequency objects (1) + adjective (1) + carrier phrase (0)^a^	2	40
5	Mixed frequency objects (1) + 2 adjectives (2) + carrier phrase (0)	3	60
6	Two mixed frequency objects (0)^b^ + preposition (1)	1	20
7	Mixed frequency objects (2) + adjective (2) + preposition (1)	5	200
8	One sentence picture description. CIU:WC	100	100 for each of 20 stimuli, averaged.

*Note.* CIUs = correct information units, described in Treatment Stimuli section; WC = word count.

a Once the carrier phrase was mastered (Level 3), it was expected in all future levels and not assigned additional points.

b Mastery of the preposition was the focus of training at this level.

Level 1 consists of a high-frequency word deck in which requesting the object by name is the goal (e.g., “horse”). Level 2 is a low-frequency word deck in which requesting the object by name is required (e.g., “anchor”). In Levels 1 and 2, the production of one word was required, and percent correct was scored as the percentage of words produced accurately, such that a naïve listener would understand the meaning out of context. For example, “horz” would be considered an appropriate substitution for “horse” but not “torse” or “horser.”

The same criteria were used for higher levels, but more words from the sentence were taken into consideration. Level 3 is composed of mixed frequency objects requiring the carrier phrase (e.g., “Do you have the anchor?”). The carrier phrase “Do you have the” was counted as correct or incorrect and was weighted equally with the noun production. Again, percent correct was calculated. Level 4 uses a mixed frequency object deck requiring a single adjective to differentiate between nouns (e.g., frying pan vs. dust pan). Level 5 also uses mixed frequency objects, but additional stimuli are included so that the request must incorporate multiple adjectives in order to differentiate between cards (e.g., red frying pan vs. black frying pan). At this point, the carrier phrase was established and was not counted in the calculation of the points for this level. Percent correct was calculated for the total number of adjectives and nouns produced and was counted as correct, again, if the word was both correct and intelligible to a naïve listener.

In Level 6, another mixed frequency word deck is used, this time requiring production of two objects and a preposition (e.g., “The cat is on the chair”). Level 7 is a mixed frequency word deck requiring the production of at least two objects, two adjectives, and one preposition (e.g., “The black cat is on the pink chair”). Level 8 uses a deck composed of complex pictures and requires the production of a complete descriptive sentence (e.g., “Two girls are sleeping in the canoe while a boy fishes”). There is no specifically trained sentence per picture, but the description must be adequate, such that another participant recognizes the stimulus item in question. In this last level, correct information units were calculated as a proportion of total words with a goal of 100% as in other levels. In order to ensure the potential for 100% accuracy, the word *and* was included in correct CIUs when used appropriately. This was the only deviation from the guidelines outlined by Brookshire and Nicholas (1994). Percentage of CIUs was averaged across all 10 stimulus items.
